# Effect of Digital Marketing Capabilities and Blockchain Technology on Organizational Performance and Psychology

**DOI:** 10.3389/fpsyg.2021.805393

**Published:** 2022-02-08

**Authors:** Ying Liu

**Affiliations:** School of Business, Jinggangshan University, Ji’an, China

**Keywords:** customer-linking capabilities, market-sensing, digital marketing capabilities, blockchain, organizational performance, psychology

## Abstract

Digitalization plays an integral role in the transformation of the Omni structure. This study aims to investigate the effect of digital marketing capabilities (DMCs) and blockchain technology on customer-linking capabilities (CLCs), market-sensing capabilities (MSCs), consumer-brand engagement (CBE), and firm performance in China. The study was quantitative, and a simple random sampling technique was adopted for data collection. Data were collected using a structured questionnaire, 311 questionnaires were distributed, and a 5-point Likert scale was used to collect the data from the respondents who were employed in the Omni structure industries. The research hypothesis was tested using the structural equation modeling (SEM) technique. The results have identified a significant correlation and direct effect between DMCs, CLCs, MSCs, and firm performance. Remarkably, the effect of DMCs on CBE is significant. The mediating effect of MSCs and CLCs is significant between the relationship of DMCs and firm performance. The organization performance in the Omni structure depends on how well the DMCs have been employed. The DMCs influence MSCs, CLCs, and CBE. Henceforth, this study contributes by analyzing the role of DMCs in blockchain technology.

## Introduction

The digital and social media techniques have transformed the way consumers access and use information by replacing traditional marketing methods. Digital marketing is reliant on digital platforms and technologies to promote modern marketing strategies to attract potential customers (Chaffey and Smith, 2017). The rapid application of technology, plus changing market trends and procedures, reflects the importance of digitalization (Blut et al., 2018). There is extensive research that shows that customers use smartphones in day-to-day activities, which leads to improvement in their purchasing habits and the overall shopping process (Grewal et al., 2017). In fact, the convergence of smartphone and web-based online shops and supermarket chains as a way of creating the Omni-channel customer experience has been recognized as a new means to resolve the changing direction of retail organizations as agents who promote both physical and digital market infrastructure (Brynjolfsson et al., 2015), and China is not exempted in following this trend.

In Asia, though, several businesses have failed to introduce Omni-channel strategies that meet consumer expectations and function properly (Hosseini et al., 2018; Cao and Tian, 2020). Moreover, this has contributed to a situation where retailers are constantly faced with the difficulty of effectively transforming their business models, which affects the value of their Omni-channel retailing (Massa et al., 2017). As a result, implementing a digital platform must integrate all aspects of corporate performance to ensure that businesses in China achieve full performance quality (Chai-Arayalert and Suttapong, 2020).

On the contrary, implementing digitalization involves several challenges, such as lacking digital capabilities (Kane et al., 2015), uncertain performance outcomes, and the need for transformation of organizational functioning. Further studies on digital marketing capabilities (DMCs) and firm performance are required (Wang, 2020). Furthermore, the COVID-19 crisis has expanded the possibilities and significance of DMCs for businesses (Pedersen et al., 2020; Singh et al., 2020). To stop the spread of the virus, many governments introduced social distancing on a mass scale, so that during the COVID-19 crisis, digital sale platforms were prioritized by many consumers (Herhausen et al., 2015). However, according to Payne et al. (2017), research on consumer-brand engagement (CBE) in the context of Omni-channel marketing is limited.

Therefore, the objective of this study is to explore the role of DMCs concerning market-sensing capabilities (MSCs), customer-linking capabilities (CLCs), and CBE to improve the retailing performance of the firms in China. Second, in this study, firm performance is addressed in the context of the Omni industry. Furthermore, despite exhaustive research, the researcher could not find any study that examined the impact of DMCs on CBE in the Omni structure context. Thus, this study fills a gap in the existing research on this topic.

This study aims to make many contributions. First, it responds to a call for research to fill the gap in marketing skills between available and needed capacity to deal with the dynamics of the digital market capability in the Omni structure context (Day, 2011). The aim is to provide a more comprehensive understanding of DMCs. Second, this article offers empirical data on the effects of the DMCs on firm performance. Finally, it contributes to the growing literature on digitalization by studying the DMCs, performance relationship *via* MSCs, CLCs, and CBE.

## Theoretical Background

### Digital Marketing Capabilities

Digital marketing capabilities represent the capabilities of the firm that empower it to adapt its resource formations and build new skills in dealing with stakeholder communication in real-time (Kane et al., 2015). They tend to improve the efficiency of social networking and market analysis concerning stakeholders.

Additionally, DMCs often relate to the relational skills needed to take advantage of the benefits of digitalization (Wang, 2020). They must intrinsically be adaptive so that decisions can be flexible and versatile. Likewise, Teece (2012) recognized the difference between ordinary routine capabilities and dynamic capabilities that facilitate companies to adapt to quickly evolving environments. Common skills ensure that the existing business procedures run smoothly. For instance, the capability of the international systems (IS) is common to support the activities of the supply chain. It operates and builds an efficient network for all the ISs. However, DMCs are dynamic capabilities, which are capable of causing the change in the time of resource combination processes (Eisenhardt and Martin, 2000). They are also useful when there is a need to handle the issues related to the business firm and its stakeholders. Similarly, DMCs assist firms to digitally coordinate and manage relationships with suppliers, customer linking, and channel members. As a result, the firm performance improves (Rai et al., 2006). Moreover, it can be observed that DMCs enhance CBE through changing positive behavior for online shopping (Scheinbaum, 2016). In this scenario, the role of DMCs could be influential on CBE (Farook and Abeysekara, 2016).

### Customer-Linking Capabilities

Customer-linking capabilities are the true essence of maintaining a relationship with potential customers and strengthening the bond with existing customers to attract new ones (Vorhies et al., 2011) in order to increase customer lifespan value (Persson and Ryals, 2014). Sharing product information with consumers, receiving consumer requests, working with customers to handle demand, providing an order placement scheme, communicating order status with consumers during order preparation, and the product distribution process are all examples of customer linkage (Leeflang et al., 2014). In this dynamic age, CLCs are crucial because customers look for fast replies to their questions and instant shipments.

Furthermore, since CRCs are often correlated with the inclusion of technology solutions, it is important to consider how and when to use digital technologies (IT) to sustain customer relationship management (CRM) (Wang et al., 2013; Singh et al., 2020). Furthermore, as a channel for collecting information on issues encountered by clients, after-sales services play an important role in customer linkage. A quick reaction to clients is possible, thanks to the smooth and efficient contact among all stakeholders in the supply chain, including customers, after-sales canters, country sales companies, and suppliers. Moreover, CLCs enable digital marketing activities, strategies development, and execution capability, which relate to the overall ability of a firm to succeed (Chinakidzwa and Phiri, 2020a). Perhaps, the CLC is a valuable addition to the process of the firms. This distinctive capability enhances the marketing activities, potentially contributing to the success of the business. Given the statement, the research states that the proliferation of new technologies has expanded the reach of the firms by creating an interactive online environment for the consumers (Clohessy et al., 2019), thereby boosting the revenue of the firm. Undoubtedly, this new paradigm has made businesses invest in new technologies for strengthening the CLCs. Given the illustration, the emergence of new technology solutions has significantly altered the marketing activities of the firms, thereby enhancing the ways the firms can engage with the customers, thus achieving the success of the firms.

### Market-Sensing Capabilities

Market-sensing capabilities represent the potential of the firm in recognizing opportunities and foreseeing market changes. Furthermore, MSCs are also defined as the extent to which a firm can actively and purposefully observe the changes in the general environment in terms of the needs of the customer, technological advancement, and modifications in competitor strategies (Miocevic and Morgan, 2018). In addition, Morgan et al. (2012) further explained MSCs as being responsive to the changing demands of the consumers, strategies, and tactics of the competitors, emerging developments in the market structure, and wide-ranging markets and future trends.

The evolution of new technology has changed the business environment by providing numerous business opportunities to firms. Significantly, the interactive technology platforms have made the marketers sense the brand opportunities, thus building long-term consumer-brand relationships. In the illustration, the study states that MSCs improve the performance of the firms by enhancing managerial capabilities around the consumer needs and demands (Sánchez-Gutiérrez et al., 2019). In parallel, the marketing literature suggests that firms use MSCs to gain valuable knowledge for improving the performance of the firms (Cao et al., 2019). Given the articulation, the research suggests firms generate knowledge for targeting the prospective population, thereby cultivating a strong relationship with the consumers. In particular, marketers use external knowledge for responding to market opportunities (Likoum et al., 2020). The MSCs encourage innovativeness, brand engagement, thus achieving superior organizational performance. Firms owning these capabilities substantially improve the competitiveness of the firms, thus strengthening organization performance. In support, the study shows that MSCs allow the marketers to realize opportunities, eventually leveraging the dynamic capabilities to enhance the performance of the firms (Laaksonen and Peltoniemi, 2018). Perhaps, the MSC plays an integral role in configuring the market capabilities into innovative performance and competitiveness of the firms.

Undoubtedly, MSCs are essential, as marketing analysis can provide comprehensive information on the current and future needs of the consumers. Additionally, firms must assess opportunities in data-rich environments to have the best solutions based on the available resources (Wedel and Kannan, 2016; Singh et al., 2020). Manufacturing companies can easily find design defects and offer improved after-sales service to their customers (Coreynen et al., 2017), which eventually improves the performance of the firm.

### Consumer Brand Engagement

Both academics and practitioners have paid careful attention to the concept of consumer engagement. Specifically, in the broader sense, CBE has also received much attention in the literature (Hollebeek, 2011). CBE represents the psychological condition of customers, which is mainly dependent on the interactive and co-creative experience with the main brand in the market (Brodie et al., 2011). In addition, several outcomes have been generated by the CBE, such as market effects, consumer effect, brand effect, content effect, and product effect (Barger et al., 2016).

The brand effect consists of the perceived quality of the product, consumer awareness about the brand, loyalty, and associations (Graffigna and Gambetti, 2015), while product effects are comprised of the behavior of consumers toward the product and the frequency of purchase, which is based on the experience of the customer (Ismagilova et al., 2020). The content effect covers the attitudes of the consumers. It activates toward the brand through means such as customer ratings and reviews, re-sharing intentions, and brand-related content (Herhausen et al., 2015). Consumer effects are based on social capital, self-prediction behavior, and consumer power (Brandão et al., 2019; Singh et al., 2020). Last, market effects represent changes in market-level strategies in terms of changes in retailing channels and advertisements, purchase intentions, and conversion rates (Dolbec and Fischer, 2015).

In particular, the adaptation of new retailing channels (i.e., online platforms) has improved the consumer experience, thus enhancing CBE (Essamri et al., 2019). The emerging technologies (i.e., digital marketing) have altered the consumer experience by creating value for the targeted customers (Gielens and Steenkamp, 2019). Thus, echoing this fact, the innovation from businesses has nurtured the firm marketing performance by building strong customer engagement (Önder and Treiblmaier, 2018). Therefore, it has become vital for firms to meet the changing demand of the customers for building long-term customer-brand relationships (Langaro et al., 2018). Research shows that consumer prefers brands that care about their needs, assist them in the decision-making process, and reject the brands that do not value the interest of the consumers (Dodoo, 2018). In such a situation, CBE plays an integral role in developing customer-brand interaction (Abdullah et al., 2018; Hollebeek et al., 2019), thus gaining business success.

### Firm Performance

Generally, performance indicates how well an organization is achieving its goals, missions, and values (Gandhi et al., 2017; Khalil et al., 2021). A firm performance, on the other hand, refers to the method of determining the efficiency and efficacy of a particular operation or action (Neely et al., 1995). Marketing performance measurement is the assessment of “the relationship between marketing activities and business performance” (Clark and Ambler, 2001).

Previous studies have claimed that firm performance is represented by some common financial and non-financial measures. Financial measures include net profit, return on assets, inventory turns, net income before tax, inventory management performance, productivity ratio, financial liquidity, market share, quality performance, and before gross tax margin (Gandhi et al., 2017; Sarfraz et al., 2021). In contrast, non-financial measures cover market share, competitive position, performance, quality improvement, and innovation performance.

**FIGURE 1:**
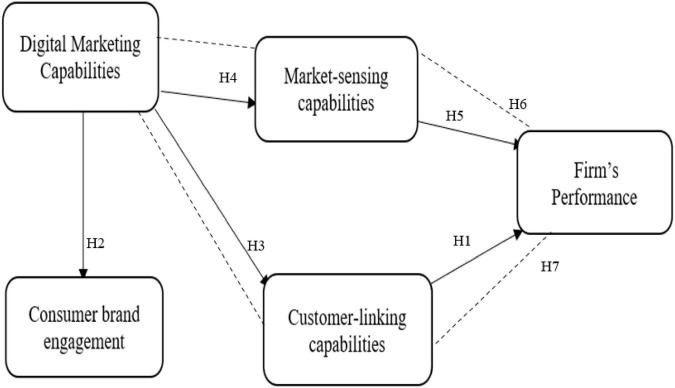
Conceptual model.

Measuring a firm performance is the key factor in sustaining the efficiency and effectiveness of its management (Demirbag et al., 2006). Improvement is difficult to achieve without evaluating the current performance first. Therefore, measuring how the use of organizational resources in terms of different offline and online channels affects business efficiency is essential for organizational performance enhancement (Sharma and Gadenne, 2010; Shah et al., 2021). Based on the previous literature and the gaps identified, this study proposes the following hypothesis. Figure 1 shows the independent, dependent, and mediating variables of the study.

H1: CLCs have a positive effect on firm performance.
H2: DMCs have a positive effect on CBE.
H3: DMCs have a positive effect on CLCs.
H4: DMCs have a positive effect on MSCs.
H5: Market sensing capabilities have a positive effect on firm performance.
H6: CLCs have a mediating effect on the relationship between DMCs and firm performance.
H7: MSCs have a mediating effect on the relationship between DMCs and firm performance.

## Research Methodology

This study is quantitative with the approach being cross-sectional. Data were collected from the target population of China who was employed in the Omni structure industries. This study uses employees from service sectors as a sample. The study selected proportionate strata sampling and convenient sampling. After making each stratum, the researcher has visited those conveniently available employees and posted the questionnaires whose addresses were accessible.

According to the data collection procedure, 10 companies were selected, and questionnaires were distributed electronically; 380 self-administered questionnaires were distributed to the employees from service sectors. This technique is also confirmed in the study by Shah (2009). Moreover, some questionnaires were received back through courier from the stated firms of China. Out of 380 questionnaires that were returned, 311 (81%) questionnaires were valid.

### Measurement

In this study, a 5-point Likert scale was used to measure the items. DMCs were measured with six items adopted by Wang (2020). CBE was measured with six items adopted by Hollebeek et al. (2014). MSCs were measured with six items adopted by Lindblom et al. (2008). CLCs were measured with five items adopted by Cao and Tian (2020), and firm performance was measured with five items (Croteau and Bergeron, 2001; Kearns and Sabherwal, 2006).

## Results

The next move is to look at convergent validity, which describes the degree of a positive association between measurements or metrics with the same construct (Hair et al., 2016). Researchers looked at the average variance extracted (AVE) and indicators of outer loading (Hair et al., 2016). The AVE threshold value is 0.50 or greater, suggesting sufficient convergent validity, or half of the variance of metrics explained by latent constructs (Hair et al., 2017a). The meaning of AVE less than 0.50 indicates that more variation exists in item error than in the variance explained by the build (Hair et al., 2016). Generally, scales or markers with outer loading between 0.40 and 0.70 can be excluded (Hair et al., 2017b). The larger the outer loadings, the more frequent the build indicators are; this is often known as indicator reliability (Hair et al., 2017b). As a result, the convergent validity of the current analysis was determined by analyzing the AVE values and outer loadings. The findings show that both of the AVE values of the constructs are greater than 0.50. As shown in Table 1, convergent validity has been identified.

**TABLE 1:** Internal consistency reliability.

Construct	Cronbach’s alpha	rho_A	Composite reliability	Average variance extracted (AVE)
CBE	0.744	0.852	0.828	0.545
CLC	0.748	0.902	0.824	0.522
DMC	0.871	0.875	0.912	0.721
FP	0.925	0.927	0.943	0.769
MSC	0.902	0.905	0.927	0.719

The discriminant validity is defined as extends to a construct that is distinct from the other constructs by empirical standards (Hair et al., 2016). In other words, the indicators of construct which are theoretically distinct to other constructs are also distinct by empirical standards. The established discriminant validity means that a construct that captures the phenomena is distinct from other constructs in the same model.

The approach commonly used for the assessment of discriminant validity is the Fomell-Larcker criterion. This approach compares the square root of the AVE values with the correlations of a latent variable (Hair et al., 2016). Therefore, the square root of the AVE values should be higher than its correlation with any other construct in the model (Henseler et al., 2009). Hence, in this study from the Fornell-Larcker criterion, the square root of the AVE values of the construct is greater than the highest correlation with any other constructs, so it is concluded that the discriminant validity has been established, and the results are shown in Table 2.

**TABLE 2:** Discriminant validity.

	CBE	CLC	DMC	FP	MSC
CBE	0.738				
CLC	0.591	0.723			
DMC	0.784	0.574	0.849		
FP	0.385	0.338	0.382	0.877	
MSC	0.441	0.469	0.503	0.449	0.848

### Structural Model

**FIGURE 2:**
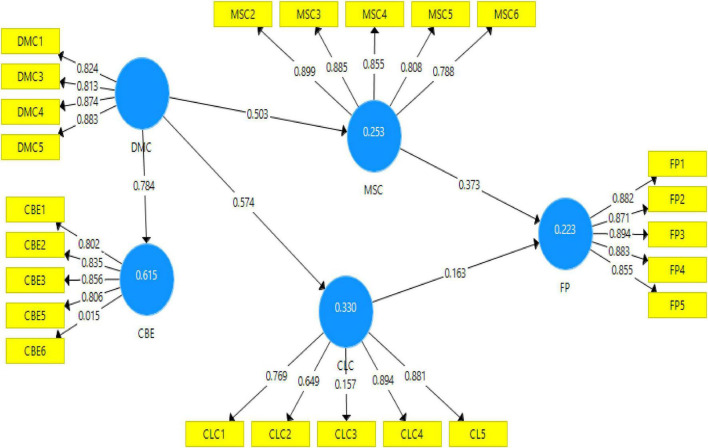
Measurement model.

**FIGURE 3:**
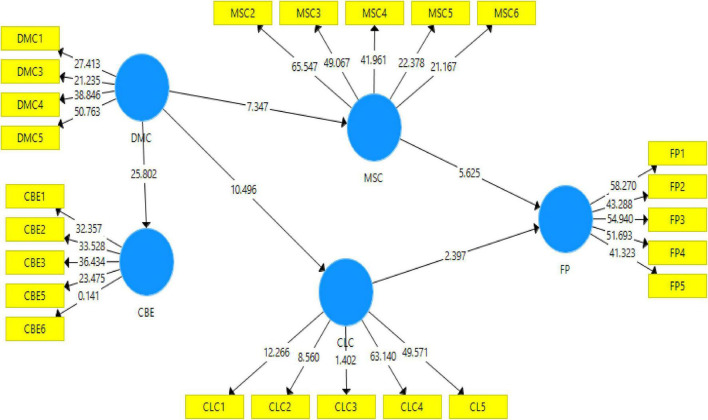
Structural equation model.

According to Hayes technique, the study is used to examine the effect of mediating variables on the relationship between the independent and dependent variables by assessing the structural or inner model. In the PLS-SEM, the key criteria for structural model assessment are the assessment of predictive relevance (*Q*^3^) (Hair et al., 2017b). Therefore, Figure 2 shows the assessment measurement model, and Figure 3 shows the structural equation modeling (SEM) evaluated structural models.

#### Direct Relationship

Customer-linking capabilities are positively related to the firm performance. Table 3 demonstrates a significant and positive relationship between CLCs and the firm performance (β = 0.163, *t* = 2.397, *p* = 0.017), thus, hypothesis 1 (H1) is supported. Hypothesis 2 (H2) predicted that DMCs are positively related to CBE. Table 3 shows a significant and positive relationship between DMC and CBE (β = 0.784, *t* = 25.802, *p* = 0.000), thus, H2 is accepted. Hypothesis 3 (H3) predicted that DMCs are positively related to CLCs. Table 3 describes a significant and positive relationship between DMC and CLCs (β = 0.574, *t* = 10.496, *p* = 0.000), thus, H3 is accepted. Hypothesis 4 (H4) predicted that DMCs are positively related to MSCs. Table 3 demonstrates a significant and positive relationship between DMCs and MSCs (β = 0.503, *t* = 7.347, *p* = 0.000), thus, H4 is accepted. Similarly, hypothesis 5 (H5) predicted that MSCs are positively related to firm performance. Results in Table 3 demonstrate a significant positive relationship between MSCs and firm performance (β = 0.373, *t* = 5.625, *p* = 0.000), thus, H5 is accepted.

**TABLE 3:** Direct relationship.

Hypothesis	Relationship	Original sample (O)	*T* statistics (| O/STDEV|)	*P* values	Decision
H1	CLC → FP	0.163	2.397	0.017	Accepted
H2	DMC → CBE	0.784	25.802	0	Accepted
H3	DMC → CLC	0.574	10.496	0	Accepted
H4	DMC → MSC	0.503	7.347	0	Accepted
H5	MSC → FP	0.373	5.625	0	Accepted

Hypothesis 6 (H6) predicted that the mediating effect of CLCs between DMCs and firm performance is significant (β = 0.094, *t* = 2.145 > 1.96, *p* = 0.032 < 0.05), therefore, H6 is accepted. Hypothesis 7 (H7) predicted that the mediating effect of MSCs between DMCs, and firm performance is significant (β = 0.187, *t* = 4.152 > 1.96, *p* = 0.000 < 0.05), therefore, H7 is accepted (see Table 4; Chinakidzwa and Phiri, 2020b).

**TABLE 4:** Indirect relationship.

Hypothesis	Relationship	Original sample (O)	*T* statistics (| O/STDEV|)	*P* values	Decision
H6	DMC → CLC → FP	0.094	2.145	0.032	Accepted
H7	DMC → MSC → FP	0.187	4.152	0	Accepted

In Table 5, *Q*^2^ shows that the value of CBE is 0.301, CLC is 0.143, firm performance is 0.158, and MSC is 0.167. Hair et al. (2016) argued that the model has predictive relevance if the *Q*^2^ value is greater than zero.

**TABLE 5:** Predictive relevance.

Construct	SSO	SSE	*Q*^2^ (=1 − SSE/SSO)
CBE	1,290.00	902.121	0.301
CLC	1,290.00	1,105.33	0.143
DMC	1,032.00	1,032.00	
FP	1,290.00	1,085.96	0.158
MSC	1,290.00	1,074.83	0.167

## Discussion

The aim of the study was to examine the effect of DMCs and CLCs on firm performance through the mediating effect of MSCs and CLCs. Furthermore, the study examines the effect of DMCs on CBE.

Hypothesis 1 predicted that CLCs are positively related to firm performance. It demonstrated a significant and positive relationship between CLCs and firm performance (β = 0.163, *t* = 2.397, *p* = 0.017), and the results are similar to that suggested by Leeflang et al. (2014), thus, H1 is supporting. H2 predicted that DMCs are positively related to CBE. It demonstrated a significant and positive relationship between DMCs and CBE (β = 0.784, *t* = 25.802, *p* = 0.000). The results are similar to that suggested by Wang (2020), thus, H2 is accepted. H3 predicted that DMC is positively related to CLCs. It demonstrated a significant and positive relationship between DMCs and CLCs (β = 0.574, *t* = 10.496, *p* = 0.000), and the results are similar to that suggested by Wang et al. (2013), thus, H3 is accepted. H4 predicted that DMC is positively related to MSCs. It demonstrated a significant and positive relationship between DMCs and MSCs (β = 0.503, *t* = 7.347, *p* = 0.000), and the results are similar to that suggested by Wedel and Kannan (2016), thus, H4 is accepted. Similarly, H5 predicted that MSCs are positively related to firm performance. It demonstrated a significant positive relationship between MSCs and firm performance (β = 0.373, *t* = 5.625, *p* = 0.000), and the results are similar to that suggested by Coreynen et al. (2017), thus, H5 is accepted. Furthermore, H6 predicted that the mediating effect of CLCs between DMCs and firm performance is significant (β = 0.094, *t* = 2.145 > 1.96, *p* = 0.032 < 0.05), and the results are similar to that suggested by Singh et al. (2020), thus, H6 is accepted. H7 predicted that the mediating effect of MSCs between DMCs and firm performance is significant (β = 0.187, *t* = 4.152 > 1.96, *p* = 0.000 < 0.05), and the results are similar to that suggested by Chinakidzwa and Phiri (2020b), thus, H7 is accepted.

## Conclusion

Progressed technologies have significantly extended the e-business process for value creation. The innovative advances have altered the dynamic of brand marketing by exclusively providing new products and services to the customer. The emerging technologies have reshaped the marketing discipline by promoting advanced marketing techniques (e.g., applications, software, and infrastructures) for leveraging the worldwide reach of businesses to satisfy the demands of the modern marketplace. In this process, blockchain technology has compelled the technological communication media to strengthen the bond with CLCs, MSCs, and CBE. Today, the novel technologies have allowed the marketers to penetrate deeper into the new marketspace (i.e., digital marketing), thus sensing the changing customer demands. Perhaps, this dynamic market engagement (i.e., CBE) uses modern technologies to enhance consumer involvement, thereby improving the firm performance. However, blockchain technology potentially fosters the firm marketing activities by incrementing innovation, MSCs, subsequently empowering the consumer-centric paradigm.

In fact, this study has examined the important inter-relationships between DMCs, CBE, CLCs, and MSCs in the context of China. According to the findings of the study, the role of DMCs is very important in enhancing both the firm performance and CBE. Furthermore, CLCs and MSCs as mediators played a very important role in maximizing the firm performance, specifically in the Omni industry structure.

### Theoretical and Practical Implications

The study revealed two gaps in marketing capabilities: the insufficient current and ideal marketing capabilities between managers. Second, a knowledge gap was discovered, and the contribution of a significant division of DMCs and transformations in industrial service firms extended the scholarly knowledge to rectify this. The COVID-19 crisis heightened the importance and opportunities of DMCs, and this study guides researchers and policymakers in this endeavor.

### Study Limitations and Future Research

This study has some limitations. First, the study was conducted only in the context of China. In the future, another sample could be taken from other ASEAN countries such as Malaysia, Singapore, and Indonesia. Second, this study did not focus on a single retail firm, even though doing so would allow for a more finely tuned study of industry-specific DMCs regarding the firm performance. Third, this study used a quantitative approach to test the hypothesis. In the future, both quantitative and qualitative approaches could be employed. For international marketing and the Omni-channel structure, there is still a need for more conceptualization of DMCs.

## Data Availability Statement

The original contributions presented in the study are included in the article/supplementary material, further inquiries can be directed to the corresponding author.

## Ethics Statement

Ethical review and approval was not required for the study on human participants in accordance with the local legislation and institutional requirements. Written informed consent for participation was not required for this study in accordance with the national legislation and the institutional requirements.

## Author Contributions

The author confirms being the sole contributor of this work and has approved it for publication.

## Conflict of Interest

The author declares that the research was conducted in the absence of any commercial or financial relationships that could be construed as a potential conflict of interest.

## Publisher’s Note

All claims expressed in this article are solely those of the authors and do not necessarily represent those of their affiliated organizations, or those of the publisher, the editors and the reviewers. Any product that may be evaluated in this article, or claim that may be made by its manufacturer, is not guaranteed or endorsed by the publisher.
